# Characteristics of retinal detachment associated with atopic dermatitis

**DOI:** 10.1186/s12886-021-02135-7

**Published:** 2021-10-11

**Authors:** Youlim Lee, Woo Kyung Park, Rae-Young Kim, Mirinae Kim, Young-Gun Park, Young-Hoon Park

**Affiliations:** 1 Department of Ophthalmology, Yonsei University College of Medicine, Seoul, Republic of Korea; 2 Department of Ophthalmology and Visual Science, College of Medicine, The Catholic University of Korea, Seoul, Korea; 3 Catholic Institute for Visual Science, College of Medicine, The Catholic University of Korea, Seoul, Korea; 4 Department of Ophthalmology, Seoul St. Mary’s Hospital, College of Medicine, The Catholic University of Korea, 222 Banpo-daero, Seocho-gu, Seoul, Republic of Korea

**Keywords:** Atopic dermatitis, Retinal detachment, Cataract surgery

## Abstract

**Background:**

To examine the characteristics of rhegmatous retinal detachment (RRD) associated with atopic dermatitis.

**Method:**

Medical records of 2257 patients who underwent RD surgery at this clinic between 2008 and 2018 were retrospectively reviewed. Among them, 61 patients who were diagnosed as AD were assigned into the experimental group and 100 patients who did not have AD were randomly selected and assigned into the control group.

Demographics, characteristics of detachment, initial operative method, and prognosis after surgery were investigated as main outcomes. Additionally, in pseudophakic RD patients, the period between the cataract surgery and onset of RD was measured.

**Result:**

Postoperative VA and prognosis were significantly worse and bilateral involvement of RD was more common in the atopy group than in the control group. (*P value = 0.005, 0.001 each)* Characteristics of retinal breaks were different between the two groups. Additionally, the risk of developing RD within 1 year after cataract surgery was significantly higher in pseudophakic patients of the atopic group than in the control group. (*P value = 0.013*) However, there was no significant difference in mean preoperative VA or refractive index between the two groups.

**Conclusion:**

Our results show that in atopic patients, RD occurs at a young age with different characteristics compared to non-atopic patients. Atopic RD has a poor visual prognosis. Thus, it requires careful management. Furthermore, the risk of developing RD within 1 year after cataract surgery is higher in atopic patients. Therefore, it is important to perform regular and extensive check-up after cataract surgery for atopic patients.

## Introduction

Atopic dermatitis (AD) is a common disease with a worldwide prevalence of 15-25% in children and 7% in adults. Similar prevalence of AD has been reported also in Korea [1]. According to a previous study, AD patients have 2 to 12 times more ocular diseases such as dry eye, keratoconjunctivitis, keratoconus, retinal detachment (RD), glaucoma, and cataract than non-atopic patients [2].

.RD is a pathological condition in which the retina is detached from the inner wall of the eyeball. The annual incidence of RD is about 12 in 100,000 persons, and if this condition continues, permanent retinal atrophy occurs, resulting in blindness and atrophy of the eyeball [3]. It has been shown that RD occurs 3.22 times more in AD patients than in the general population [2] with a frequency of 8-10% in patients with AD between 10 and 30 years of age, and lead to a poorer prognosis in AD patients than in a non-AD population [4, 5].

.If the retina is detached, the retina is not supplied with nutrients and the function of photoreceptors gradually decreases [6]. Therefore, vireo-retinal surgery is required in most cases of RD. The pars plana vitrectomy (PPV) is one of the most effective surgical method for the treatment of RD with high anatomical success rate, the mean postoperative reattachment rate being 93.3% [7]. The risk factors for low functional and anatomic outcomes after primary RD surgery are as follows: more than 6 days of visual loss, macular involvement and the size of detachment area, and independency from surgical procedures (buckling or vitrectomy) chosen to repair the detachment [4]. It is known that usage of steroids may influence the inflammatory and proliferative components of proliferative vitreoretinopathy (PVR), by reducing the breakdown of the blood-retinal barrier [8–10].

Although causes of atopy-related RD have not been elucidated yet, it has been suggested that RD might be caused by atopic edema induced by allergies or blood vessel abnormalities known to be associated with atopic dermatitis [2], or diseased vitreous that can cause retinal break due to immune response [11]. The most widely accepted theory is t*h*at frequent rubbing of the eye in AD patients might lead to traction and retinal tear of the anterior vitreous, resulting in RD. One study has reported that the incidence of RD is about 8 times higher in people with frequent eye rubbing [12]. Moreover, it has been noted that RD associated with AD is more frequent in bilateral involvement than RD associated with congenital anomaly or inflammation, or high myopia. Atopic RD is also associated with a higher risk of surgical failure due to PVR [13]. PVR is believed to be the leading cause of failure of RD surgery with accounts for 75% of retinal re-detachment surgeries [14].

.Although the risk of RD in AD patients is known to be high, there have been few studies on clinical characteristics of atopic RD. Especially, the risk of developing RD after cataract surgery in AD has not been reported yet. Therefore, the objective of this study was to investigate the frequency and clinical characteristics of RD in AD patients in a single institution after a relatively long follow-up period.

## Methods

Medical records of 2258 patients who underwent RD surgery in Seoul St. Mary’s Hospital (Seoul, Republic of Korea) between 2008 and 2018 were retrospectively reviewed. A total of 61 (2.7%) patients had AD. They were assigned to the experimental group. Of 2197 patients who did not have AD, 100 patients were randomly selected for the control group. Patients with RD due to trauma, exudate, or macular hole were excluded from this study. AD was diagnosed by a dermatologist based on the ‘Diagnostic criteria for Korean atopic dermatitis’.

All patients analyzed in this study underwent a complete preoperative evaluation, which included a comprehensive history taking, best-corrected visual acuity (BCVA) measurement, slit-lamp microbioscopy, fundus examination with a contact wide angle viewing lens (Superquad 160, Volk) that could evaluate range, type, and location of retinal break, optical coherence tomography to confirm macular involvement, and presence of proliferative vitreoretinopathy (PVR) (those classified as Grade 3 or above according to the classic classification of the Retina Society) [13]. Optical coherence tomography (OCT) was done using a swept-source OCT device (DRI Triton, Topcon, Tokyo, Japan).

Break types had six categories; retinal dialysis, horseshoe tear, retinal tear (as tear except horseshoe tear), hole with lattice, hole without lattice, and unknown (if the type of break was unclear). The location of the break had five categories; superotemporal, inferotemporal, superonasal, inferonasal, and unknown.

BCVA and refraction (RK-F1®, Canon, Tokyo, Japan) were compared before surgery Refraction was excluded if it occurred in a pseudophakic state. In eyes with a history of cataract surgery, the mean period between the cataract surgery and the diagnosis of RD was recorded.

The range of the area with RD involvement was defined as the number of quadrants containing RD.

In all patients, the surgeon decided the surgical procedure by considering fundus and lens status, age, location of tear, and PVR. The surgery was performed using the procedure of silicone sponge (506-silicone; Labtician, Oakville, ON, Canada) buckling, 23 or 25 gauge pars plana vitrectomy (Accurus surgical system; Alcon Laboratories, TX, USA), or both. Additional sub-retinal drainage, air-gas exchange, silicone oil implantation, and laser photocoagulation were performed if necessary.

To evaluate anatomical and functional outcomes, postoperative BCVA and recurrence rate of RD were investigated at 6 months and the last visit after surgery.

In subjects with RD for both eyes, each eye was recorded separately. For two cases of the atopy group, the opposite eye was a localized RD and cured only with the use of a barrier laser. Those two subjects were excluded from analysis. Among evaluated bilateral patients, if the operation of the other eye was performed in another hospital, the other eye was excluded from this study; one case in each group.

### Statistical analysis

Logarithm of the minimum angle of resolution (log MAR) was used for VA analysis. VA values for counting fingers, hand movements, and light perception were then assigned ratios of 0.01, 0.001, 0.0005, respectively. A Mann-Whitney U test was used to compare demographics, surgical prognosis and refractive status between the two groups. Chi-square test was used to compare initial surgery method and demographics. All statistical analyses were performed using SPSS statistical software for Windows, version 25.0 (SPSS, Chicago, IL, USA). Statistical significance level was set at *P* < 0.05.

## Results

In this study, there were a total of 2258 patients who had RD surgery in our clinic between 2008 and 2018. Of these, 61 patients had AD and it accounts for 2.7% of total RRD patients.

Demographic characteristics of study participants are summarized in Table 1.

**Table 1:** Demographic and clinical characteristics of the study participants

	**Atopy**	**Non-atopy**	***P* value**
**Demographics**			
No. of patients (eyes)	61 (71)	100 (100)	
Sex – male:female (%)	39:22 (64:36)	63:37	0.811
Mean age (year)	23.08 ± 10.24	52.29 ± 15.48	0.000*
Involved eye - OD:OS (%)	37:34 (52:48)	50:50	0.785
Preop VA (Log MAR)	1.14	0.96	0.294
Number of bilateral involved patients: n (%)	13 (21%)	1 (1%)	0.000*
Lens status; phakic: pseudophakic (%)	43:28 (60:40)	74:26	0.063

Values are presented as mean ± SD unless otherwise indicated

*VA* Visual acuity

*p-value by Mann-Whitney U test and chi-square test

Of these 61 patients with AD, 13 had bilateral RD. Two of them had localized RD that was treated with barrier laser only. For that reason, they were not included in this study. Additionally, one patient received RRD surgery for the opposite eye from another hospital. That eye was excluded from the experimental group. Thus, a total of 71 eyes were included in the atopy group.

Of the 100 control patients, only one patient had bilateral RD. The other eye of this patient received RD surgery from another hospital. That eye was not included in this study. Therefore, a total of 100 eyes were included in the control group.

The rate of bilateral RD was 21% in the atopic group, which was significantly higher than that (1%) in the control group (*P value = 0.000*).

There were 39 (64%) males and 22 (36%) females in the atopy group, and 63 (63%) males and 37 (37%) females in the non-atopy group (*P value = 0.811*).

**Fig. 1:**
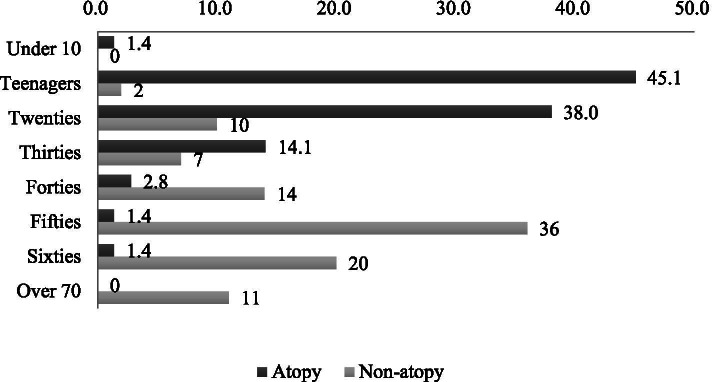
Age of patients. (%)

The mean age was 23.08 ± 10.24 years in the atopy group, which was significantly (*P value = 0.000*) younger than that (52.29 ± 15.48 years) in the control group (Table 1, Fig. 1).

In the atopy group, 37 had RD in the right eye and 34 had RD in the left eye. In the non-atopy group, 50 had RD in the right eye or the left eye, showing similar rates in both groups. There was also no statistically significant difference in the lens status (phakic or pseudophakic) between the two groups either.

The mean spherical equivalent before surgery was − 3.09 ± 0.392 in the atopic group and − 3.56 ± 0.372 in the control group, showing no significant difference between the two groups (*P value = 0.953*, Table 2).

**Table 2:** Refractive status before surgery

**Refractive status**	**Atopy**	**Non-atopy**	***P* value**
Spherical equivalent (Mean ± SD)	−3.09 ± 0.392	− 3.56 ± 0.372	0.953
Hyperopia	1 (1%)	7 (7%)	
Emme to -3D	31 (43.7%)	44 (44%)	
Myopia (3D to-8D)	25 (35.2%)	30 (30%)	
High myopia (over -8D)	5 (7%)	19 (19%)	0.005*

Values are presented as mean ± SD unless otherwise indicated

Values are presented as n (%)

*p-value by Mann-Whitney U test

Initial operative method was also analyzed. Results are shown in (Table 3). PPV alone was performed for 21% (15 eyes) of the atopy group and 59% (59 eyes) of the control group. Buckling alone was performed for 17% (12 eyes) of the atopy group and 31% (31 eyes) of the control group. Encircling alone was performed for 24% (17 eyes) of the atopy group and 7% (7 eyes) of the control group. Encircling and ppV were performed at the same time as an initial surgery for in 38% (27 eyes) of the atopy group and 3% of the control group, showing significant difference between the two groups.

**Table 3:** Initial method of surgery in each group

	**Atopy**	**Non-atopy**
Initial OP method		
Pars plana vitrectomy	15 (21%)	59 (59%)
Buckling	12 (17%)	31 (31%)
Encircling	17 (24%)	7 (7%)
ppV + Encircling	27 (38%)	3 (3%)
With phacoemulsification	11(15%)	17 (17%)
With Pars plana lensectomy	4 (6%)	10 (10%)
	15 (21%)	27 (27%)

Values are presented as n (%)

*p*-value by chi-square test

As the initial surgery, “encircling” was performed for 62% of the atopy group and 10% of the control group, showing difference between the two. In the control group, the most used operation method was ppV alone.

In this study, fifteen (21%) eyes of atopic patients received cataract surgery and RD surgery at the same time. Phacoemulsification in 11 eyes and pars plana lensectomy (ppL) in 4 eyes were performed. In the control group, 27 eyes had cataract surgery together; with phacoemulsification (17 eyes) or and ppL (10 eyes).

Preoperative log MAR VA was 1.14 for the atopy group and 0.96 for the control group, showing no significant difference between the two groups. Postoperative log MAR VA was 0.80 for the atopic group, which was significantly (*P value = 0.05*) poorer than that (0.34) for control group. Preoperative macular-off ratio showed no statistically significant difference between the two groups. PVR rate was 35% for the atopy group, which was significantly (*P value = 0.000*) higher than that (12%) for the control group. Reoperation rate was 35% for the atopy group, which was also significantly (*P value = 0.001*) higher than that (10%) for the control group. Therefore, PVR and reoperation rate were shown to be significantly higher in the atopy group (Table 4).

**Table 4:** Pre-operative visual acuity and macula, PVR status & post-operative visual & surgical prognosis of each group

	**Atopy**	**Non-atopy**	***P* value**
Pre op VA (Log MAR)	1.14	0.96	0.294
Post op VA (Log MAR)	0.80	0.34	0.005*
Macula ON: OFF (%)	34:37 (48:52)	44:56	0.615
Rate of PVR (%)	35%	12%	0.000*
Re-operation rate (%)	30%	10%	0.001*

*p-value by Mann-Whitney U test and chi-square test

Data of RD involving quadrant are present in Table 5. RD was limited to one quadrant in 6 (8.5%) eyes in the atopy group and 9 (9%) eyes in the control group. RD involved two quadrants in 56.3% of the atopy group and 66% of the control group. It involved three quadrants in 25.4% of the atopy group and 18% of the control group. RD involved all four quadrants in 9.9% of the atopy group and 7% of the control group. These differences between the two groups were not statistically significant (*P value = 0.213*).

**Table 5:** Number of involved quadrants of RD

	**Atopy**	**Non-atopy**	***P* value**
Involved quadrant			0.213
1	6 (8.5%)	9	
2	40 (56.3%)	66	
3	18 (25.4%)	18	
4	7 (9.9%)	7	

Values are presented as n (%)

*p*-value by Mann-Whitney U test

The location of the retinal breaks was also analyzed. Results are shown in (Table 6). In the atopy group, retinal breaks were found in the superotemporal and inferotemporal areas as 34 and 30%, respectively. In the control group, breaks were mainly in the superotemporal area (50%). The type of retinal breaks was also analyzed (Table 7). Horseshoe tear and retinal tear were observed in 30 and 28% of the atopic group and 35 and 27% of the control group, respectively. Retinal dialysis was more frequent in the atopy group (16% in the atopy group vs. 2% in the control group, *P value = 0.000*), whereas lattice hole was more observed in the control group (8% in atopy group vs. 21% in control group, *P value = 0.000*).

**Table 6:** Location of breaks by fundus quadrant

	**Superotemporal**	**Inferotemporal**	**Superonasal**	**Inferonasal**	**Unknown**	***P* value**
Atopy	24 (34%)	21 (30%)	6 (8%)	10 (14%)	10 (14)	
Non-Atopy	50 (50%)	21 (21%)	21 (21%)	6 (6%)	2 (2%)	
						0.001*

Values are presented as n (%)

*p-value by chi-square test

**Table 7:** Types of retinal break

	**Retinal dialysis**	**Horseshoe tear**	**Retinal tear**	**Lattice hole**	**Hole without lattice**	**Unknown**	***P* value**
Atopy	11 (16%)	21 (30%)	20 (28%)	6 (8%)	6 (8%)	7 (10%)	
Non-Atopy	2 (2%)	35 (35%)	27 (27%)	21(21%)	14 (14%)	1 (1%)	
							0.000*

Values are presented as n (%)

*p-value by chi-square test

In this study, twenty-eight (39%) out of 71 eyes in the atopy group, and 26 (26%) out of 100 eyes in the control group had undergone a cataract surgery before the onset of detachment. RD occurred at 1.93 ± 0.48 years after the cataract surgery in the atopy group and at 4.86 ± 0.90 years after the cataract surgery in the control group, showing a significant difference between the two. (*P value 0.002*, Table 8). Notably, RD occurred within 6 months after cataract surgery in 9 (32%) of 28 eyes in the atopy group and 4 (15%) of 26 eyes in the control group (*P value = 0.154*). RD within 1 year after cataract surgery was significantly more common in atopy group; 17 (60%) of 28 eyes in the atopy group and 7 (26%) of 26 eyes in the control group (*P value = 0.013*).

**Table 8:** Mean period after cataract surgery in patients who underwent cataract surgery before

	**Atopy**	**Non-atopy**	***P* value**
Mean period after cataract surgery (years)	1.93 ± 0.48	4.86 ± −0.90	0.002*
Within 1 year; n (%)	17 (60%)	7 (26%)	0.013*
Within 6 month; n (%)	9 (32%)	4 (15%)	0.154

Values are presented as mean ± SD unless otherwise indicated

*p-value by Mann-Whitney U test

## Discussion

Characteristics of RD associated with atopic dermatitis were analyzed in this study. Although some studies have already reported the characteristics of atopic RD, this is the first report that compares characteristics of atopic RD with those of non-atopic RD. Moreover, we compared the incidence of RD associated with cataract surgery, tear type or location in an atopy group and non-atopy group.

In Korea, the prevalence of atopic dermatitis is 2.2% in the total population, 6.9% in those under 18 years of age, 0.9% of those over 18 years of age [15]. In this study, 2.7% of all RD patients had atopy. And in both groups the number of male patients was 1.7 times more than that of female patients. Although the prevalence of atopy itself is not significant different by gender in previous studies, male patients were more common than female patients of RD in this study. This result was similar to one study from Japan, showing that the incidence of atopy was not different by gender but the rate of RD was nearly doubled in males [2]. In the control group, male patients were more than females, consistent with a previous study showing that men were at a higher risk for RD than women regardless the presence of AD [2].

.In the control group, the incidence rate of RD by age was the highest in those aged 50-60 years and the second highest in the age group of 20s. Likewise, a previous study has reported bimodal distribution of RD [16]. However, in the atopy group, most patients were teenagers or in their 20s. The mean age of onset was 52 years in the control group and 23 years in the atopy group, showing significant difference between the two (*P value = 0.000*).

Bilateral RD accounts for 5-10% of total RD in a previous study [2]. In th present study, 21% of subjects in the atopy group and 1% in the control group showed bilateral involvement of RD. The rate of bilaterality was significantly higher in the atopy group than in the control group.

There was no significant difference in the mean preoperative refractive index between the two groups. However, the proportion of high myopia patients with − 8 diopters or more was 7% in the atopic group, which was significantly lower than that (19%) in the control group. (*P value = 0.005*).

There was no significant difference in RD involving quadrant or preoperative VA between the two groups. However, postoperative VA was significantly poorer in the atopy group (log MAR VA 0.80) than in the control group (log MAR VA 0.34), *(P value = 0.005*). Although VA before surgery was similar, the prognosis after surgery was significantly worse in the atopy group. This might thought to be due to inflammation caused by atopy itself, delayed wound healing, or increased PVR.

In the control group, the superotemporal area was the most common location of the retinal tear, followed by inferotemporal area and superonasal area similar rates for these two areas. In the atopy group, the ratio of unknown was 14%, higher than that (2%) in the control group. Most tears noted in the atopy group were found in the temporal area. Tears showed similar rates in superotemporal and inferotemporal areas. These results suggests that RD might occur due to frequent rubbing of the lateral side of eyes in the atopy group. However, the temporal area was also common in the control group, further studywith more subjects might be needed to confirm this finding.

The incidence of RD showed no significant difference between right and left eyes. This result suggests that right-handedness and left-handedness might play no role in the incidence of RD.

Interestingly, the type of tear was characteristically different between the two groups. Retinal dialysis was found in 16% of the atopy group, which was significantly higher than that (2%) of the control group. A previous study has shown that slapping of the eye can acts as a trauma, resulting in retinal dialysis [17].

.Retinal dialysis refers to disinsertion of the retina from the vitreous base. It is an uncommon cause of RD, accounting for 8 to 17% of all RD cases. A previous study has suggested that retinal dialysis might be associated with trauma in many cases [18, 19]. Trauma caused by eyelid rubbing could be associated with atopic RD. Horseshoe tear and retinal tear had a similar incidence rates in both groups. However, lattice hole was more frequently found in the control group.

PVR is a disease process that involves proliferation of ectopic cell sheets in the vitreous and/or periretinal area, causing periretinal membrane formation and traction, in patients with RRD [2]. Previous studies have shown that PVR occurs in 5–10% of all RRD cases. PVR is implicated in re-detachment after surgery in 75% of cases, remaining a major barrier to successful repair of RD [17]. In our study, the PVR rate was 35% in the atopy group and 12% in the control group, showing a statistically significant difference between the two *(P value = 0.000*). As mentioned above, RD with atopy is generally thought to be associated with eye rubbing, which often leads to poor peripheral retinal and to more occurrences of PVR.

Previous studies have shown that about 13% of patients needed reoperation after a single surgery of RD [20]. As mentioned above, it is known that the anatomical success rate of PPV reaches 93.3% [21]. In this study, the reoperation rate was 30% in the atopy group, which was three times higher than that of the control group (10%), showing a statistically significant difference (*P value = 0.001*) between the two.

Despite macular-off rate and preoperative VA were similar between two groups, vitrectomy and encircling together as an initial operative method was performed more in the atopy group than in the control group (38% vs. 3%) The surgical method of vitrectomy and encircling together is usually performed for patients with severe PVR or retinal reattachment due to retinal dialysis. Despite these aggressive treatments, both recurrence rate and postoperative visual prognosis were poorer in the atopy group than those in the control group, consistent with a previous study [20].

.At the time of RD occurence, pseudophakic cases accounted for 40% in the atopic group and 26% in the control group. The mean time between the cataract surgery and onset of RD was 1.93 ± 0.48 years in atopy group and 4.86 ± − 0.90 years in control group, showing statistically significant difference between the two groups. Especially, RD within 1 year after cataract surgery occurred in 60% of the atopic group and 26% of the control group. This suggests that the risk of RD might be increased after cataract surgery in atopy patients, especially at up to 1 year post surgery.

In phakic eyes, the movement of the vitreous body might be limited due to the lens. Thus, the progression of RD could be suppressed to a certain extent. However, after a cataract surgery, these mechanisms might be broken and be more susceptible to RD development due to trauma of eyelid rubbing. In addition, atopic patients could be more vulnerable to RD due to their specific immune reactions and the resulting degeneration of the vitreous body.

This study has some limitations such as its retrospective design, the small sample size and the relatively short follow up. Therefore, further randomized clinical trials with a longer follow-up and a larger sample are highly demanded to clarify the differences in the demographics, clinical findings, and management of atopy-related RD and its complications. In addition, steroids were used as intravitreal triamcinolone only with the PPV surgery in both group. The effect of steroids was not considered in comparison with other surgical methods, so further studies may be needed.

In conclusion, atopic patients had RD at a younger age with poorer prognosis due to high incidence of PVR or recurrence. Therefore, extensive treatment and management are needed for these patients. Moreover, atopic patients had a much higher risk of RD after cataract surgery than controls. Thus, careful examination should be performed regularly for atopic patients after a cataract surgery to achieve the best patient outcomes.

## Data Availability

The datasets during and/or analyzed during the current study are available from the corresponding author on reasonable request.
